# Snare deroofing technique-assisted endoscopic retrograde direct cholangioscopy for difficult cannulation of Type III duodenal papilla

**DOI:** 10.1055/a-2651-0156

**Published:** 2025-08-27

**Authors:** Shan-Shan Hu, Xiao-gang Liu, Xiao Hu, Yun-Chao Yang, Wei-Hui Liu

**Affiliations:** 1 Department of Gastroenterology and Hepatology, Sichuan Provincial People’s Hospital, School of Medicine, University of Electronic Science and Technology of China, Chengdu, China

Our team has successfully developed an innovative endoscopic retrograde direct cholangioscopy (ERDC) technique. This approach involves mounting a conical transparent cap at the tip of the cholangioscope, facilitating direct visualization during cannulation 1 2 . However, in clinical practice, the ERDC technique may still encounter cannulation difficulties, particularly in cases involving Type III elongated papillae 3 . To address this challenge, we adapted the modified endoscopic papillectomy technique 4 to improve ERDC cannulation performance in Type III difficult papillae.

A female patient presented with common bile duct stones and was scheduled to undergo the ERDC technique for stone extraction ( Fig. 1 ). Despite attempting several traditional cannulation techniques, including guidewire assistance and endoclip traction, biliary cannulation was unsuccessful ( Fig. 2 ). Papillary edema further compounded the cannulation difficulty. In order to overcome this challenge, we used a snare-assisted deroofing technique to resect the redundant mucosal layer at the papillary apex, achieving complete exposure of the bile duct orifice. Subsequently, using the ERDC technique, we successfully identified the bile duct orifice and achieved biliary cannulation ( Fig. 3 ). Stone extraction was then completed smoothly under direct visualization ( Fig. 4 ). Postoperatively, the patient recovered well and was discharged without complications ( Video 1 ).

**Fig. 1:**
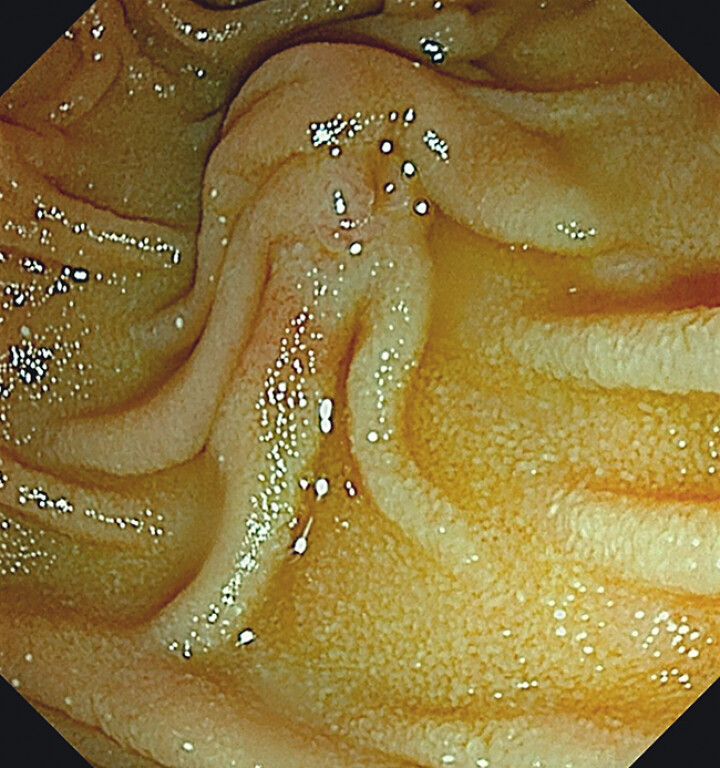
Type III duodenal papilla.

**Fig. 2:**
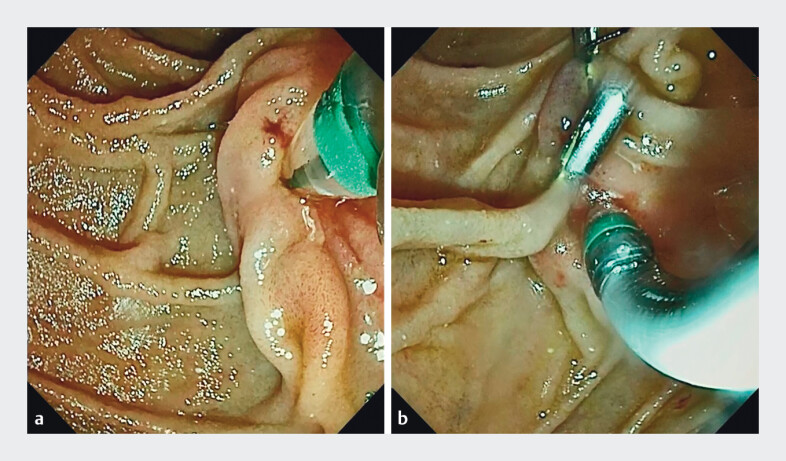
Difficult cannulation with endoscopic retrograde direct cholangioscopy. **a** Cannulation attempt using a guidewire. **b** Cannulation attempt using the metal clip traction method.

**Fig. 3:**
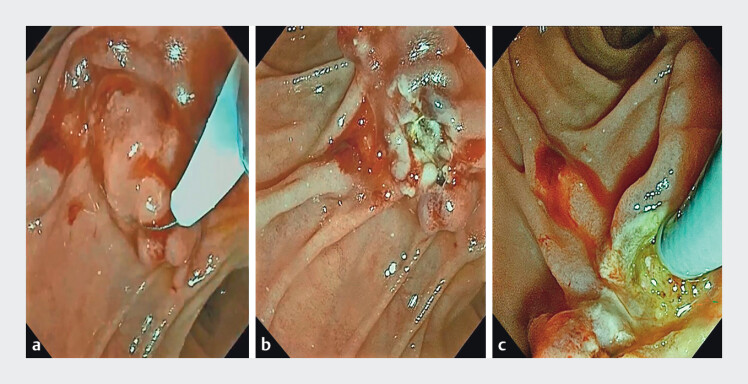
Snare-assisted deroofing to expose the bile duct opening. **a** A snare was used to perform dome resection on the excessively long mucosal layer at the apex of the papilla. **b** The bile duct orifice was fully exposed. **c** Endoscopic retrograde direct cholangioscopy resulted in successful cannulation.

**Fig. 4:**
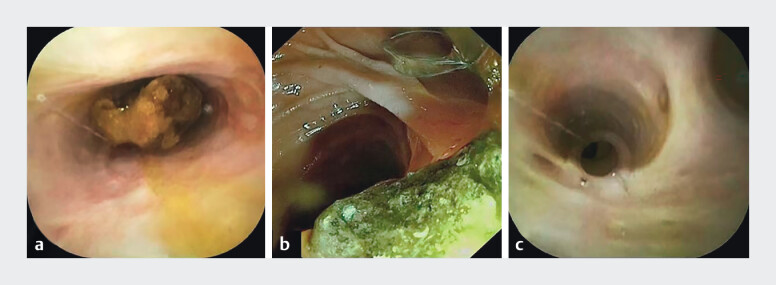
Stone extraction under endoscopic retrograde direct cholangioscopy. **a** Bile duct stones. **b** Successful stone extraction. **c** Postoperative biliary tract cleansing.

Snare deroofing technique-assisted endoscopic retrograde direct cholangioscopy for difficult cannulation of Type III duodenal papilla.Video 1

This represents the first reported successful integration of the snare deroofing technique with ERDC technology for challenging biliary cannulation of Type III papillae. This innovative approach is akin to the needle-knife fistulotomy safeguard technique used in traditional endoscopic retrograde cholangiopancreatography, serving as the ultimate protective measure for successful bile duct cannulation with ERDC technology.

Endoscopy_UCTN_Code_TTT_1AR_2AH

## Notes

Endoscopy E-Videos https://eref.thieme.de/e-videos
*E-Videos* is an open access online section of the journal *Endoscopy* , reporting on interesting cases and new techniques in gastroenterological endoscopy. All papers include a high-quality video and are published with a Creative Commons CC-BY license. Endoscopy E-Videos qualify for HINARI discounts and waivers and eligibility is automatically checked during the submission process. We grant 100% waivers to articles whose corresponding authors are based in Group A countries and 50% waivers to those who are based in Group B countries as classified by Research4Life (see: https://www.research4life.org/access/eligibility/ ). This section has its own submission website at https://mc.manuscriptcentral.com/e-videos .
